# A Network Pharmacology Study to Explore the Underlying Mechanism of Safflower (*Carthamus tinctorius L.*) in the Treatment of Coronary Heart Disease

**DOI:** 10.1155/2022/3242015

**Published:** 2022-05-14

**Authors:** Qingwen Meng, Huajiang Liu, Haolin Wu, Ding shun, Chaoling Tang, Xinyin Fu, Xingyue Fang, Yiqian Xu, Bocen Chen, Yiqiang Xie, Qibing Liu

**Affiliations:** ^1^Deparment of Vasculocardiology, The First Affiliated Hospital of Hainan Medical University, Haikou 570100, China; ^2^Department of Pharmacology, Hainan Medical University, Haikou 570100, China; ^3^Department of Pharmacy, The First Affiliated Hospital of Hainan Medical University, Haikou 570100, China; ^4^College of Traditional Chinese Medicine, Hainan Medical University, Haikou 570100, China

## Abstract

Safflower has long been used to treat coronary heart disease (CHD). However, the underlying mechanism remains unclear. The goal of this study was to predict the therapeutic effect of safflower against CHD using a network pharmacology and to explore the underlying pharmacological mechanisms. Firstly, we obtained relative compounds of safflower based on the TCMSP database. The TCMSP and PubChem databases were used to predict targets of these active compounds. Then, we built CHD-related targets by the DisGeNET database. The protein-protein interaction (PPI) network graph of overlapping genes was obtained after supplying the common targets of safflower and CHD into the STRING database. The PPI network was then used to determine the top ten most significant hub genes. Furthermore, the DAVID database was utilized for the enrichment analysis on Gene Ontology (GO) and Kyoto Encyclopedia of Genes and Genomes (KEGG). To validate these results, a cell model of CHD was established in EAhy926 cells using oxidized low-density lipoprotein (ox-LDL). Safflower was determined to have 189 active compounds. The TCMSP and PubChem databases were used to predict 573 targets of these active compounds. The DisGeNET database was used to identify 1576 genes involved in the progression of CHD. The top ten hub genes were *ALB, IL6, IL1B, VEGFA, STAT3, MMP9, TLR4, CCL2, CXCL8*, and *IL10*. GO functional enrichment analysis yielded 92 entries for biological process (BP), 47 entries for cellular component (CC), 31 entries for molecular function (MF), and 20 signaling pathways, which were obtained from KEGG pathway enrichment screening. Based on these findings, the FoxO signaling pathway is critical in the treatment of CHD by safflower. The in vitro results showed that safflower had an ameliorating effect on ox-LDL-induced apoptosis and mitochondrial membrane potential. The western blot results showed that safflower decreased Bax expression and acetylation of FoxO1 proteins while increasing the expression of Bcl-2 and SIRT1 proteins. Safflower can be used in multiple pathways during CHD treatment and can exert anti-apoptotic effects by regulating the expression of Bax, Bcl-2, and SIRT1/FoxO1 signaling pathway-related proteins.

## 1. Introduction

According to the American Heart Association's Heart Disease and Stroke Statistics (2020 edition), cardiovascular disease is a growing public health concern worldwide [1]. According to the China Cardiovascular Health and Disease Report 2020 Edition, the prevalence of cardiovascular disease in China is on the rise, with an estimated 330 million people suffering from the disease. Cardiovascular disease is the primary cause of death in both urban and rural areas. As the primary risk factor for cardiovascular death, coronary heart disease (CHD) has long caused serious harm to families and society. Interventional therapy and medicine are the main treatment strategies for patients with CHD. Although these methods are rapid and effective, they are also accompanied by side effects, such as postoperative rebleeding and adverse reactions.

Traditional Chinese medicine (TCM) is multitargeted and multidirectional and is therefore used in the treatment of CHD. Dehydrated flowers of the safflower plant (*Carthamus tinctorius L.*), which belongs to the subclass Dicotyledoneae and family Compositae, are commonly used in clinics to treat CHD, hypertension, and cerebrovascular diseases, as well as to enhance blood flow and remove blood blockage [2]. Modern pharmacological studies have shown that safflower has vasodilating, anticoagulant, antihypertensive, antioxidant, anti-inflammatory, and analgesic effects [3]. Previous studies have demonstrated that safflower inhibits the JNK1/2-NF-*κ*B pathway, which prevents lipopolysaccharide (LPS)-induced TNF signaling activation and cell death in H9c2 cardiomyoblast cells [4]. However, it is difficult to determine the mechanism of safflower in the treatment of CHD. The active target mechanism of its effective chemical components particularly requires further exploration.

The network pharmacology approach is based on the biochemical construction of a drug-component-disease target network. It explores the relationship between the target and pathway, while clarifying the molecular mechanism. This technique delineates the holistic and systematic features of TCM, and it has significant benefits over traditional approaches in terms of comprehending and depicting comprehensive mechanisms. It is an approach used to discover active components, validate the efficacy and mechanisms of drug actions and interactions, confirm the beneficial active components of the metabolic process, and investigate prototype compounds. It has recently been used to demonstrate potential mechanisms of TCM therapy for illnesses such as CHD and diabetes. By creating a network model, network pharmacology may be utilized to understand the links between components and pathways.

**Figure 1:**
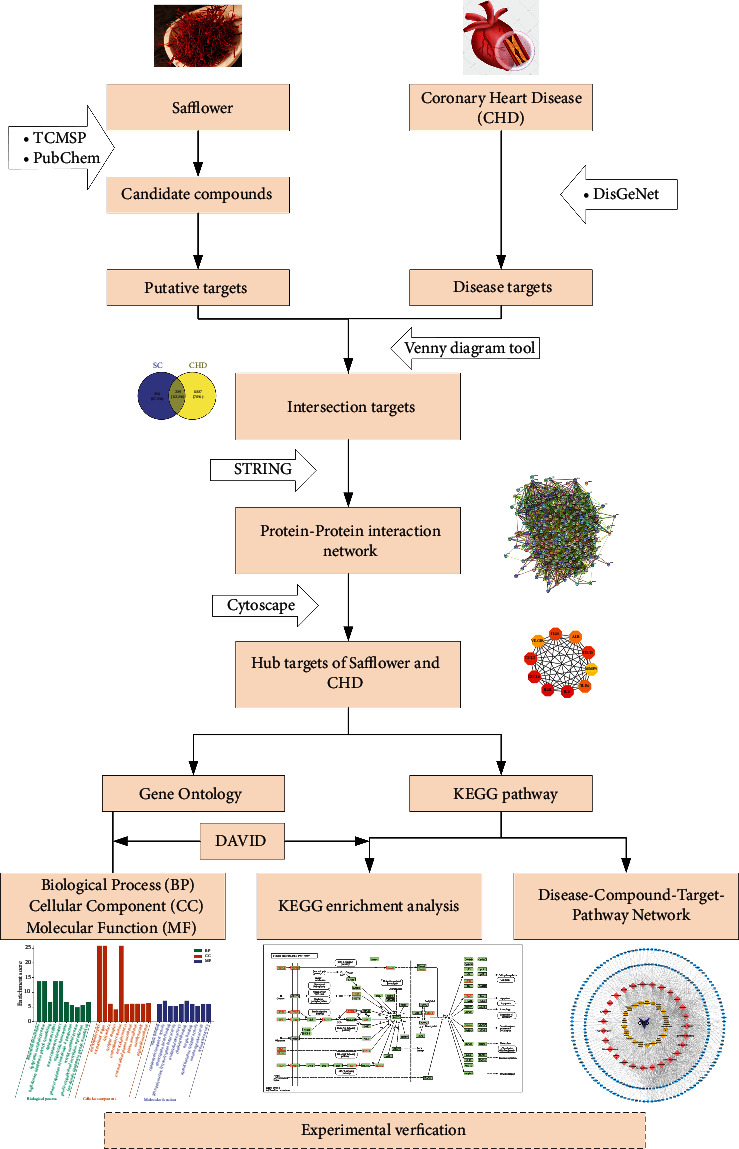
The overall process flow of the study.

In this study, the potential mechanism of safflower in CHD therapy was evaluated using a network-based systematic analysis. A comparative analysis was also performed to identify overlapping genes. Furthermore, overlapping genes and hub nodes were used to build protein-protein interaction (PPI) and module networks, which were then analyzed for Gene Ontology (GO) and Kyoto Encyclopedia of Genes and Genomes (KEGG) pathway enrichment. We aimed at investigating the potential therapeutic targets of safflower in the treatment of CHD, which would serve as a foundation for further research into the pharmacological mechanism of safflower. Subsequently, we used in vitro experiments to validate the predicted molecular mechanism of safflower in coronary heart disease therapy as determined by network pharmacology. The results of these experiments provide a foundation for the clinical application of safflower in CHD patients. The research process is illustrated in Figure 1.

## 2. Materials and Methods

### 2.1. Network Pharmacology

#### 2.1.1. Construction of the Effective Compounds of Safflower

The effective compounds of safflower were obtained by screening the Traditional Chinese Medicine Systems Pharmacology Platform (TCMSP) database (http://lsp.nwu.edu.cn/tcmsp.php). TCMSP is an open database that contains the oral bioavailability (OB) of nearly 500 Chinese herbal medicines and their drug similarity (DL), blood-brain barrier (BBB), intestinal epithelial pass permeability (Caco-2), and other reference values. The compounds contained in safflower were screened using the TCMSP database, with oral bioavailability set to OB ≥ 30% and drug likeness set to DL ≥ 0.18.

#### 2.1.2. Screening for the Targets of Safflower

Typically, medicinal components interact with relevant biological pathways via their targets. To estimate the targets of compounds found in safflower, details on the molecular structure of the bioactive constituents in safflower were obtained from the TCMSP and PubChem databases (https://pubchem.ncbi.nlm.nih.gov/). The top 100 target genes corresponding to each compound were searched from the chemical-gene co-occurrences in the literature module of the PubChem database. Subsequently, genes corresponding to the targets of the safflower compounds were determined using the STRING database (https://www.string-db.org/).

#### 2.1.3. Collection of Targets Related to the Pathogenesis of CHD

Enter the keyword “coronary artery disease” in the DisGeNet database to search for the corresponding target(https://www.disgenet.org/). DisGeNET is an exploratory platform containing genes associated with human diseases and their comorbidities. According to Score gda> 0.1, targets related to the pathogenesis of CHD were selected.

#### 2.1.4. Identification of Common Targets of CHD and Safflower

A Venn diagram depicting the intersection of safflower targets and CHD targets was constructed using Venny 2.1 (BioinfoGP, Madrid, Spain). The intersecting genes were discovered by comparing safflower target genes to CHD-related pathogenesis targets.

#### 2.1.5. Disease-Compound-Target-Pathway Network Construction of Safflower in the Treatment of CHD

The Cytoscape software (version 3.6.0) was used to construct the disease-compound-target-pathway network, followed by network topology analysis. Cytoscape is a visualization software that can integrate biomolecular interaction networks, data on high-throughput gene expression, and other molecular details.

#### 2.1.6. Construction and Analysis of Protein-Protein Interaction (PPI) Network Diagram

To explain the relationship between projected targets and other human proteins, a PPI network was created, which has been shown to be a viable technique for drug development. The protein-protein interaction network of potential therapeutic targets of safflower for CHD was built using the STRING database. In the designated setting, protein associations with a confidence score greater than 0.4 were selected after duplicates were removed.

### 2.2. Examination of the Hub Gene

The PPI network was imported into Cytoscape using the CytoHubba plugin, and the MCC algorithm was used. The top ten hub genes targeted by safflower to treat CHD were identified.

#### 2.2.1. Analyses of Pathway Enrichment for Core Targets

The screened targets were analyzed using the DAVID (https://david.ncifcrf.gov/) database for GO bioprocess analysis and KEGG pathway analysis. GO enrichment analysis comprises the following three aspects: biological process (BP), molecular function (MF), and cellular component (CC). The GO enrichment analysis results were selected based on *P*-values, and the top 10 pathways were selected. KEGG can be used to evaluate the target signaling pathways of medicines and identify those with the highest correlation, which is critical for determining the potential mechanism of safflower in CHD therapies. Finally, the threshold value of *P* < 0.05 was determined. Major pathways were identified, and bioinformatics software was used to map the signaling pathways. The top 20 pathways were selected based on the outcomes of KEGG pathway enrichment analysis.

### 2.3. Experimental Validation

#### 2.3.1. Cell Culture and Treatments

EAhy926 cells were purchased from the Cell Source Center, Chinese Academy of Sciences (Shanghai, China). Cells were commonly cultivated in Dulbecco's modified Eagle's medium (DMEM), 10% fetal bovine serum (FBS), and 1% penicillin/streptomycin. The cells were randomly separated into three groups during the logarithmic growth phase: the control group, oxidized low-density lipoprotein (ox-LDL) group (obtained from Yeasen Biotechnology, Shanghai, China), and safflower group. Each group had at least three replicates, and cell counting was performed to ensure that the number of cells in each group was the same. Except for the control group, the cells were cocultured with 100 µg/ml ox-LDL for 24 h and then switched to a normal medium or a safflower drug-containing medium for another 24 h, and then collected for further analysis.

#### 2.3.2. Preparation of Drug-Containing Serum

Ten male Sprague-Dawley (SD) rats (180 ± 20 g, 4 weeks old) were provided by Changsha Tianqin Biotechnology Co. Ltd. (Changsha, China). All SD rats were housed under conventional conditions (indoor temperature 24 ± 2°C, humidity 60 ± 15%, 12-h/12-h light/dark cycles) and provided with equal access to standard rodent feed and water. All experiments were conducted in accordance with China's National Guidelines for Experimental Animal Care and Use. The study was approved by the Ethical Committee of Hainan Medical University(No. HYLL-2021-140). SD rats were categorized into two groups for the preparation of safflower drug-containing serum (*n* = 5) and blank serum (*n* = 5). The typical human dose of safflower (10 g) was converted to an appropriate drug dosage for the SD male rats according to the body surface area. SD rats were given a safflower solution (1 g/kg) intragastrically via gavage for 7 days. The normal control group were given an equal volume of normal saline (NS). Blood samples were taken from the aorta one hour after the last gavage. The supernatant was extracted, filtered, and inactivated in a water bath at 56°C for 30 min, and the sera from the same group were mixed and stored at −20°C.

#### 2.3.3. Cell Viability Measurements

A Cell Counting Kit-8 was used to check the viability of cells (CCK8; Dojindo Laboratories, Kumamoto, Japan). When cells reached 80% confluence, they were treated with ox-LDL at 0, 25, 50, 100, or 200 µg/ml for 24 h. Subsequently, CCK8 solution (10 *μ*l/100 *μ*l medium) was added to the media for 2 h at 37°C in a 96-well plate. A microplate reader (EPOCH, BioTek, Winooski, VT, USA) was used to measure the absorbance of the media at 450 nm.

#### 2.3.4. TUNEL Staining

TUNEL staining is a technique used to detect DNA fragments that have formed during apoptosis (Abcam, Cambridge, UK). In each group, apoptotic cells were counted in three random fields using a fluorescent microscope.

#### 2.3.5. Detection of Mitochondrial Membrane Potential by JC-1

A JC-1 fluorescent probe was used to measure mitochondrial membrane potential according to the manufacturer's instructions (Beyotime Biotechnology, Shanghai, China). EAhy926 cells were incubated with 1 ml JC-1 at 37°C for 20 min, washed twice with PBS, and then observed and photographed using a fluorescent microscope.

#### 2.3.6. Western Blot

Cells were collected and lysed on ice in 6-well plates. BCA protein assay kits were used to measure the concentrations (Thermo Fisher Scientific, Waltham, MA, USA). SDS-PAGE was used to separate the protein samples, which were then transferred to polyvinylidene fluoride (PVDF) membranes. After blocking for 2 h at room temperature with 5% nonfat milk in Tris-buffered solution containing 0.1% Tween 20 (TBST), membranes were incubated overnight at 4°C with primary antibodies (1 : 1000) against Bax (Abcam), Bcl-2(Abcam), Sirt1 (Thermo Fisher Scientific), acetylated FoxO1 (Thermo Fisher Scientific), and *β*-actin (Abcam). After three washes, the luminescence developed, and the gel imager was exposed for imaging. The strips were analyzed for grayscale using ImageJ software (National Institutes of Health, Bethesda, MD, USA) and processed for semi-quantitative analysis, with three replicates each.

### 2.4. Statistical Analysis

All experimental data are presented as mean ± SD. Prism software was used for data processing and analysis, and one-way analysis of variance was used for comparison between groups. Significance was set to *P* < 0.05.

## 3. Results and Discussion

### 3.1. Screening for Safflower Active Compounds

We obtained 189 safflower compounds from the TCMSP database. Using the screening conditions of OB ≥ 30% and DL ≥ 0.18, 34 active compounds that met the criteria were obtained, including 6-hydroxykaempferol, baicalein, qt_carthamone, quercetagetin, beta-carotene, baicalin, beta-sitosterol, stigmasterol, kaempferol, and quercetin. Precise information on the main bioactive components is shown in Table 1.

### 3.2. Safflower for the Treatment of CHD Targets

The TCMSP and PubChem databases were used to retrieve 2527 targets from the 34 candidate components. After expelling the overlapping targets, a total of 573 targets of safflower compounds were acquired. The DisGeNET data comprise 1576 genes associated with CHD.

### 3.3. Overlapping Genes between Safflower and CHD Molecular Target Genes

**Figure 2:**
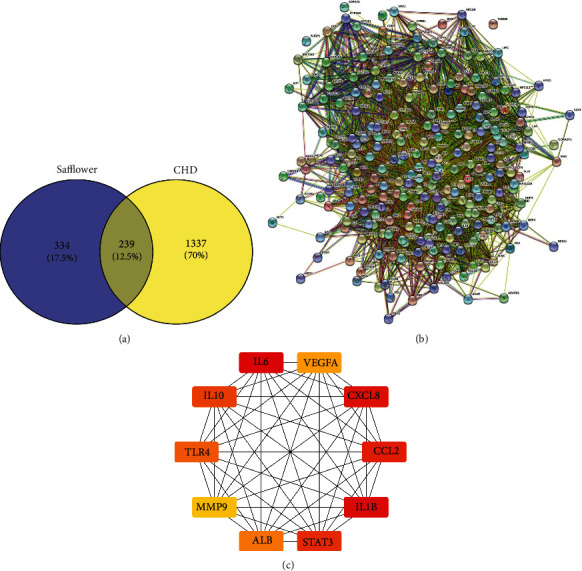
The network pharmacology of safflower in the treatment of CHD. (a) Venn diagram of safflower and CHD-related targets. (b) Protein-protein interaction network. (c) MCC algorithm analysis of the top 10 safflower hub gene networks for CHD therapy.

Using the web tool Venny 2.1, the intersection of the targets corresponding to coronary heart disease and safflower active compounds was determined using a Venn diagram. According to the findings, a total of 239 putative targets related to both CHD and safflower were identified (Figure 2(a)).

### 3.4. PPI Network Analysis

The STRING database was used to create a PPI network to further elucidate the biological mechanisms underlying the pharmacological effects of safflower on CHD. The nodes and edges in the network indicate proteins and protein-protein interactions, respectively. According to the results, the network had 239 nodes and 5819 interactions (Figure 2(b)). The average node degree was 48.7, with a local clustering coefficient of 0.634.

### 3.5. Hub Gene Scanning

The hub gene was screened in the interaction network using CytoHubba, a Cytoscape plugin. The top 10 hub genes for saffron treatment of CHD were identified using the MCC algorithm (Table 2 and Figure 2(c)), and a hub gene network diagram was created. The MCC algorithm was used to assess the top ten safflower hub gene networks for treating CHD, with the colors red and yellow indicating the network's significance.

### 3.6. Safflower Functional Enrichment Assessment in CHD

**Figure 3:**
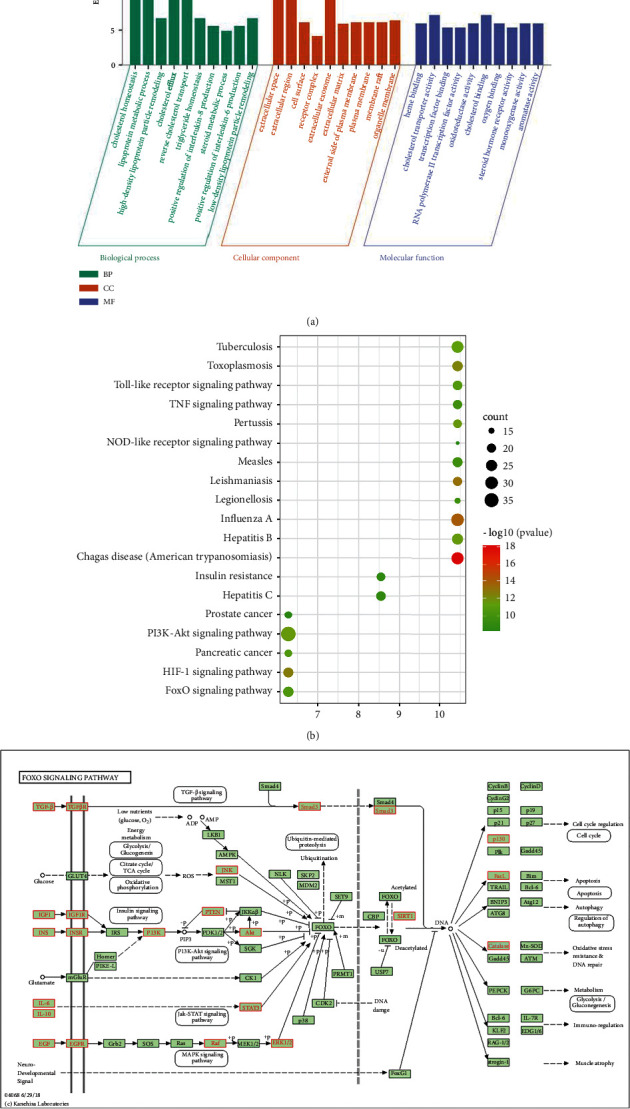
Go functional and KEGG pathway enrichment analysis. (a) GO function enrichment bubble chart of safflower in the treatment of CHD. (b) KEGG pathway analyses for the molecular signal pathway of safflower in the treatment of CHD. The node size represents the number of target genes enriched, and the node color from blue to red represents the P value from large to small. (c) FoxO signaling pathway. The targets associated with the core component-target-pathway network are represented by green rectangles.

The BP, CC, and MF annotations of the 239 selected proteins were determined using the DAVID database. GO analyses revealed 92 enriched GO terms in the category of the biological process (BP), including cholesterol homeostasis, lipoprotein metabolic process, high-density lipoprotein particle remodeling, cholesterol efflux, and reverse cholesterol transport. GO analyses indicated 47 enriched GO terms in the category of cellular component (CC), including extracellular space, extracellular region, cell surface, and receptor complex. GO analyses reflected 31 enriched GO terms in the molecular function (MF) category, including heme binding, cholesterol transporter activity, transcription factor binding, RNA polymerase II transcription factor activity, and ligand-activated sequence-specific DNA binding (Figure 3(a)).

### 3.7. KEGG Pathway Enrichment Explanation

The 239 putative targets were registered into the DAVID database for KEGG pathway enrichment analysis to explain the crucial signaling pathways targeted by safflower in CHD therapeutic approaches.  Figure 3(b) depicts the KEGG pathway enrichment analysis. The top 20 pathways were tested using a combination of count parameters and *P*-values, along with the HIF-1 signaling pathway, PI3K-Akt signaling pathway, Toll-like receptor signaling pathway, FoxO signaling pathway, and TNF signaling pathway. Based on these findings, the FoxO signaling pathway was defined as one of the most valuable signaling pathways during the treatment of CHD with safflower.  Figure 3(c) illustrates the targets for the signaling pathways that were obtained from the KEGG database (http://www.kegg.jp/kegg/mapper.html).

### 3.8. Disease-Compound-Target-Pathway Network

**Figure 4:**
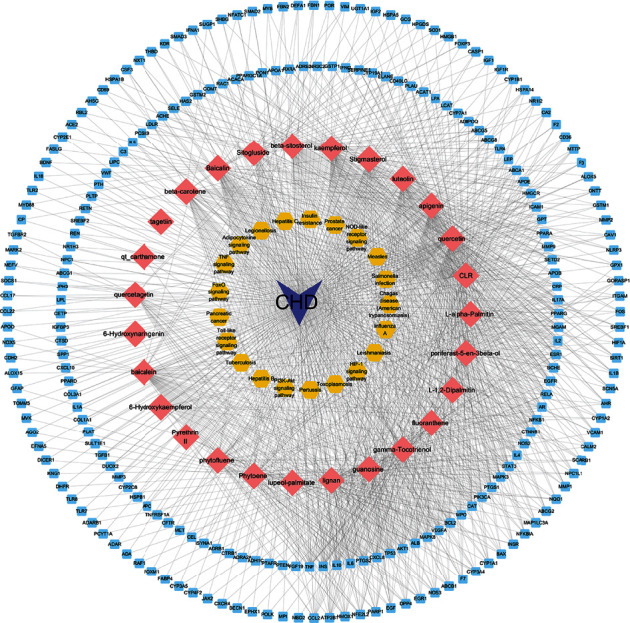
A disease-compound-target-pathway network was structured. Purple triangles symbolize CHD, pink rhombuses represent safflower chemical compounds related to common targets, and the blue squares represent chemical compound and CHD targets, while the yellow hexagons highlight key biological pathways.

To more specifically define the method of action of safflower in the treatment of CHD, a network of disease-compound-target-pathways was constructed. The network, shown in Figure 4, was constructed of disease, compound, protein targets, and pathways, with 288 nodes and 1245 edges. As for CHD, 27 components communicated with 239 target proteins and were linked to 21 pathways.

### 3.9. ox-LDL Decreased the Cell Viability of EAhy926 Cells

**Figure 5:**
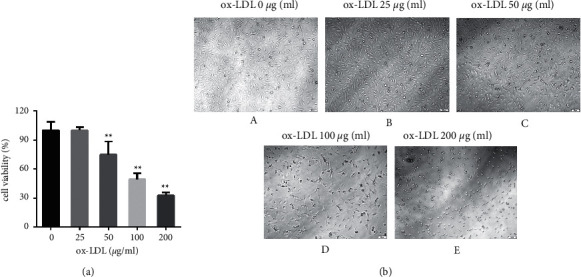
Ox-LDL decreased the cell viability of EAhy926 cells. (a) The cell survival rate of EAhy926 cells under different concentrations of ox-LDL (^*∗∗*^*P* < 0.01 vs. ox-LDL 0 *μ*g/ml group). (b) Cell morphology of EAhy926 cells under different concentrations of ox-LDL (x10).

Different concentrations of ox-LDL (0, 25, 50, 100, and 200 *μ*g/ml) were applied to EAhy926 cells for 24 h. The survival rates of each group of cells are shown in Figure 5(a) and 5(b). When the concentration of ox-LDL exceeded 50 *μ*g/ml for 24 h, cell viability decreased, as shown in Figure 5(a). At 100 *μ*g/ml, ox-LDL reduced cell viability by approximately 50%. According to the experimental results, ox-LDL 100 *μ*g/ml applied for 24 h could inhibit EAhy926 cell viability more effectively, which was used as a modeling condition for subsequent experiments.

### 3.10. Safflower Attenuated ox-LDL-Induced Apoptosis of EAhy926 Cells

**Figure 6:**
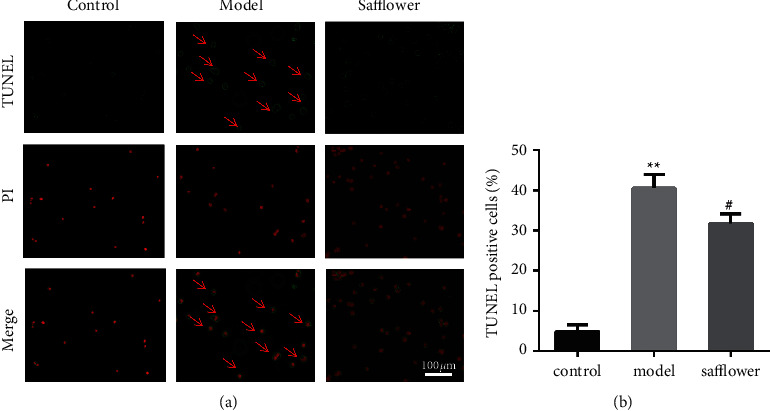
Safflower inhibits apoptosis of EAhy926 cells caused by ox-LDL. (a) TUNEL dye was applied to cells, which were then observed under a fluorescence microscope (x 20). (b) Quantitative analysis of TUNEL-positive rates (^*∗∗*^*P* < 0.01 vs. control group. #*P* < 0.05 vs. model group; *n* = 3 independent cell culture preparations).

The results of apoptosis detection by TUNEL are shown in Figure 6, and different numbers of apoptotic cells were observed in the normal, model, and safflower groups. The highest number of apoptotic cells was observed in the model group, and the apoptosis of EAhy926 cells was reduced after safflower treatment compared with the previous treatment.

### 3.11. Effects of Safflower on the Mitochondrial Membrane Potential of ox-LDL-Induced EAhy926 Cells

**Figure 7:**
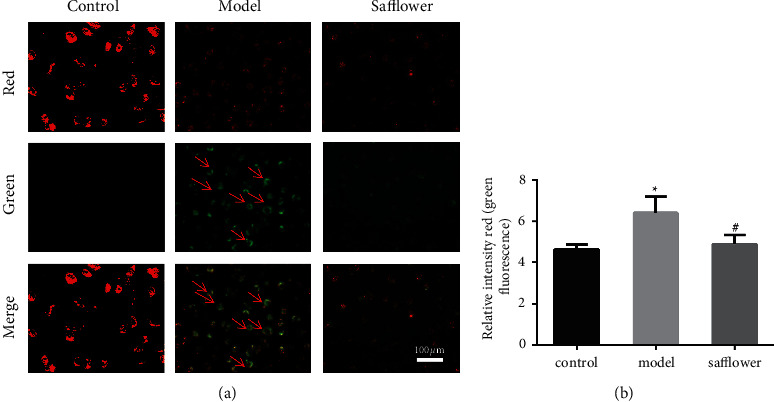
Effects of safflower on the mitochondrial membrane potential of ox-LDL-induced EAhy926 cells. (a) JC-1 was used to stain EAhy926 cells, which were then observed under a fluorescent microscope (x 20). (b) Quantitative analysis of the red/green rates after JC-1 staining; ^*∗*^*P* < 0.05 vs. control group. #*P* < 0.05 vs. model group. *n* = 3 independent cell culture preparations.

Compared to the control group, the cells in the model group had higher green and lower red fluorescence, and their mitochondrial membrane potential was significantly lower. After safflower intervention, the green fluorescence in EAhy926 cells gradually decreased, and the red fluorescence gradually increased compared to the control group. The differences were statistically significant. According to these findings, safflower may effectively resist the ox-LDL-induced decrease in mitochondrial membrane potential in EAhy926 cells (Figure 7).

### 3.12. Effect of Safflower on ox-LDL-Induced Apoptosis by Modulating the SIRT1/FoxO Signaling Pathway

The FoxO signaling pathway, which was ranked in the top 20 in the KEGG pathway analysis, plays a crucial role, and the SIRT1 protein is part of the upstream signaling pathway. SIRT1 has a deacetylating effect and is inhibited under physiological conditions; however, SIRT1 activation, increased expression, and deacetylation activity are enhanced when cells are stimulated by pathological factors such as ischemia, hypoxia, and LPS. Activated Sirt1 can deacetylate FoxO1, and deacetylated FoxO1 enters the cell plasma from the nucleus. FoxO1 exits the nucleus and loses its role in inhibiting Bax and promoting Bcl-2 expression, resulting in increased Bax expression, decreased Bcl-2 expression, and apoptosis.

**Figure 8:**
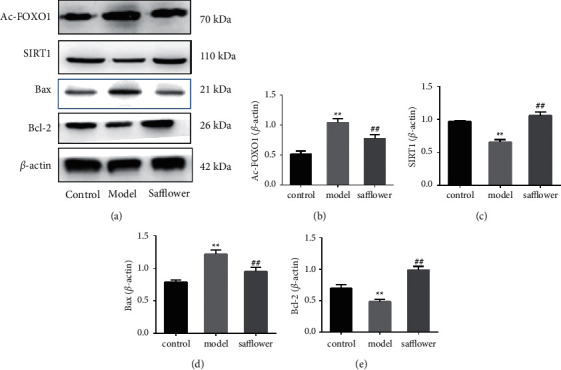
Effects of safflower on the expression of Ac-FoxO1, STIR1, and Bax, Bcl-2 in ox-LDL induced EAhy926 cells. Expression levels of Ac-FoxO1, STIR1, Bax, and Bcl-2 were detected by western blot analysis (^*∗∗*^*P* < 0.01 vs. control group; ##*P* < 0.01 vs. model group).

We therefore tested the protein expression of SIRT1 and acetylation of FoxO1 (Ac-FoxO1) in EAhy926 cells to determine how safflower affected ox-LDL-induced cardiac damage. Compared to control cells, the protein levels of SIRT1 were considerably reduced in ox-LDL-treated cells, and the protein levels of Ac-FoxO1 were significantly increased in ox-LDL-treated cells, as shown in Figure 8. However, pretreatment with safflower increased the expression of SIRT1 and simultaneously decreased the expression of Ac-FoxO1 compared to the ox-LDL group.

As demonstrated in Figure 8, ox-LDL significantly increased relative levels of Bax protein and significantly lowered relative levels of Bcl-2 protein, which was significantly inhibited by safflower pretreatment. These results indicate that the mechanism of safflower inhibition of ox-LDL-induced apoptosis in EAhy926 cells is associated with its role in regulating the SIRT1/FoxO1 pathway.

## 4. Discussion

CHD is a severe hazard to human health because it is a complex cardiovascular disease. It is characterized by ischemia, hypoxia, or necrosis of the myocardium caused by narrowing or blockage of the heart vessels due to coronary artery sclerosis or spasm. CHD is caused by a variety of factors. Single target drugs are typically ineffective for the treatment of this multifactorial disease. Furthermore, repeated or continuous high-dose administration of a single specific drug can result in drug resistance. As a result, the development of new multitarget drugs is critical. TCM has multicomponent and multitarget qualities, meaning it can target multiple biological systems to manage symptoms and treat basic problems. Safflower has been used to treat CHD for over a thousand years [5–7], but its bioactive components and processes are unknown, and the use of network pharmacology to investigate the mechanism of safflower in the treatment of CHD has not yet been reported. Therefore, we used a network pharmacology approach to investigate the active components, targets, and mechanisms of safflower in the treatment of CHD. The active substances and targets of TCM can be predicted more accurately using network pharmacology [8]. Many studies are currently being conducted to investigate the complexities of the components, targets, pathways, and mechanisms of action of herb pairings or herbal formulae using network pharmacology [9–11]. The pharmacologically active components and mechanisms involved in CHD treatment were predicted using the network pharmacology method in this study, and several proteins were verified via western blot. The components of safflower with OB ≥ 30% and DL ≥ 0.18 were found to be pharmacokinetically active, meaning they are presumably absorbed and transported throughout the body. Accordingly, the network pharmacological study of safflower revealed 34 chemicals, including *β*-sitosterol, kaempferol, quercetin, baicalein, flavoxanthin, lignan, precarthamin, carthamone, quercetagetin, and 3-methoxydaidzein that have the potential to treat CHD. *β*-Sitosterol protects the heart by modulating antioxidant enzymes via the estrogen/phosphatidylinositol 3-kinase pathway [12]. In vitro, TNF-induced apoptosis and inflammation in human umbilical vein endothelial cells are suppressed by quercetin via the NF-*κ*B and AP-1 signaling pathways [13]. Kaempferol reduces ox-LDL-induced apoptosis in human endothelial cells by increasing autophagy and inhibiting the PI3K/Akt/mTOR pathway [14]. The positive effects of Kaempferol in postmenopausal AS are linked to the PI3K/AKT/Nrf2 pathways, which are influenced by G protein-coupled estrogen receptor activation [15]. Based on these findings, baicalein has anti-inflammatory properties, most likely by activating the AMPK/Mfn-2 axis and inhibiting downstream MAPK/NF-*κ*B signaling [16]. Baicalin may prevent atherosclerosis by increasing cholesterol efflux from macrophages and delaying the production of foam cells via the PPAR-LXR-ABCA1/ABCG1 pathway [17]. According to research, baicalein, a 12/15-LOX inhibitor, can prevent myocardial I/R injury through a variety of mechanisms, and it could be used to treat acute myocardial infarction [18].

Because of the high degree of node and closeness centrality in this network, *VEGFA, STAT3, IL-6, IL-1B, MMP9, CCL2, CXCL8, TLR4, IL-10,* and *ALB* were chosen as candidate core genes. *VEGFA* is a direct target gene of miR-451 according to TargetScan and dual-luciferase reporter gene assay data and could be a new biomarker for CHD [19]. In a PDGF-BB-stimulated atherosclerosis model, the researchers discovered that circLMF1 deficiency was found to suppress cell survival, cell cycle progression, and migration, possibly via the miR-125a-3p/VEGFA or FGF1 axis [20]. In endothelial cells, fatty acid-binding protein 4 serves as a hub for angiogenic and metabolic signaling pathways. FABP4 is activated by VEGFA in endothelial cells, and inhibiting FABP4 prevents the effects of VEGFA [21]. In the PDGF-BB-stimulated atherosclerosis model, it was discovered that circLMF1 deficiency inhibited cell survival, cell cycle progression, and migration, possibly via the miR-125a-3p/VEGFA or FGF1 axis [22]. Based on its translocation, STAT3 can be classified as nuclear or mitochondrial, and both are thought to play essential roles in the development of atherosclerosis, including endothelial cell malfunction, macrophage polarization, inflammation, and immunology [23–26]. The JAK2/STAT3 pathway is intimately linked to the IL-6 cytokine family, which plays a critical role in endothelial cell dysfunction during atherosclerosis [27]. Atherosclerosis is caused by the migration of vascular smooth muscle cells (VSMCs), and matrix metalloproteinases (MMPs) play important roles in VSMC migration [28]. MMP9 is a significant regulator of the inflammatory response. The inflammatory response recruits leukocytes and produces a variety of cytokines and chemokines that increase MMP9 release, which also promotes the activation of inflammatory factors such as IL-1, resulting in a positive feedback loop [29]. When AS strikes, MMP9 levels rise, and the MMP9/NGAL complex level is closely linked to plaque vulnerability [30]. CCL2 was found in small amounts in normal arteries but was more commonly found in the intima. CCL2 and the expression of related receptors were significantly increased in diseased arteries, with relative differences between artery layers [31]. Interleukin-8 (IL-8, CXCL8) is an inflammatory agent that has been linked to the progression of atherosclerosis [32]. TLR4 is well known for its role as an important mediator of the innate immune response and has been linked to atherosclerosis initiation, progression, and plaque destabilization [33,34]. IL-10 is a pleiotropic cytokine that has been proposed as a hazard modifier for atherosclerosis [35]. One study showed that in response to inflammatory stimuli, lncRNA-CCL2 and CCL2 displayed coordinated upregulation, and their expression was associated with unstable symptomatic human atherosclerotic plaques [36]. The relationship between hypoalbuminemia, systemic albumin leakage, and soluble markers of systemic inflammation and endothelial injury has been studied in peritoneal dialysis patients, with results indicating that hypoalbuminemia is more frequently associated with inflammation and atherosclerotic disease, but little is known about the use of albumin in patients with coronary artery disease [37].

These targets are mostly connected with signal transduction pathways such as TNF, HIF-1, FoxO, Toll-like receptor, and PI3K-Akt signaling, according to our KEGG pathway analysis. As a pro-atherogenic cytokine, TNF increases the expression of cytokines and adhesion molecules, as well as the migration and mitogenesis of VSMCs and endothelial cells [38]. HIF-1 is a heterodimeric protein that belongs to the basic helix-loop-helix family and plays a major role in regulating cellular responses in low-oxygen environments. It acts on endothelial cells, vascular smooth muscle cells, and macrophages, and through cell-specific responses, it plays an essential role in the advancement of atherosclerosis [38]. Hypoxia increases macrophage glycolytic flux and upregulates proinflammatory activity in a manner that is dependent on both hypoxia-inducible factor-1 and 6-phosphofructo-2-kinase [39]. Multiple cell types and pathway cascades linked to atherosclerosis are affected by the PI3K-Akt signaling pathway. In atherosclerotic animal models, the activation of this system via vascular endothelial dysfunction lowers the formation of atherosclerotic plaques [40,41]. TNF-induced endothelial cell inflammation is mediated by METTL14. The m6A alteration of FoxO1, an essential transcription factor, was dramatically enhanced during endothelial inflammation [42]. FoxO1, IL-6, TNF-*α*, miR-27a, and miR-23a expression levels were shown to be substantially linked to the severity of stenosis in the PBMCs of patients with CAD. Compared to healthy controls, the expression of SIRT1 was shown to be significantly lower in CAD patients [43]. Macrophage polarization plays an essential role in atherosclerosis, and M1 polarization and inflammation are generated by PAR2 activation via the FOXO1-dependent pathway [44]. FOXO is a member of the forkhead transcription factor family, which plays a significant role in glucose and lipid metabolism, oxidative stress, cell cycle progression, and apoptosis. Accordingly, based on network pharmacology predictions, we mainly concentrated on the SIRT1/AC-FOXO1 signaling pathway for further exploration of the preventive role of safflower on endothelial cells. FoxO promotes atherosclerosis in endothelial cells by decreasing nitric oxide production and activating inflammatory responses [45]. SIRT1 is a histone deacetylase that is stimulated by nicotinamide adenine dinucleotide (NAD+) and is valuable in energy metabolism. SIRT1 promotes endothelial angiogenesis, homeostasis, and remodeling by regulating FoxO1 through its deacetylase activity [46]. The SIRT1/FoxO1 signaling pathway plays a major role in oxidative stress and cell pathophysiological processes such as apoptosis and metabolic regulation. SIRT1-induced deacetylation of FoxO1 suppresses apoptosis and oxidative stress by upregulating MnSOD, Bcl-2, and Bcl-XL and downregulating Bim and FasL [47]. FoxO1 is highly expressed in atherosclerotic plaques and appears to have an atheroprotective effect since FoxO1 knockdown in endothelial cells in a mouse model reduced atherosclerosis [48]. FoxO1 may contribute to the genetic predisposition to develop endothelial dysfunction and cardiovascular disease [49]. SIRT1 deficiency enhances oxidative stress, inflammation, foam cell production, and atherosclerosis progression in endothelial cells [50]. SIRT1/FoxO1 could be a promising target for preventing AS and arterial thrombosis [51]. In this study, SIRT1 expression increased, while acely-FoxO1 expression decreased in the model group, and SIRT1 expression decreased, while acely-FoxO1 expression increased after safflower intervention.

Numerous studies have found that apoptosis is important for the pathogenesis of CHD [52]. After ox-LDL stimulation, the viability of EAhy926 cells was significantly lowered, while the apoptosis rate increased, implying that ox-LDL can result in cell death. The viability of EAhy926 cells was upregulated, and the apoptosis rate was significantly lowered after safflower intervention, indicating that safflower inhibits ox-LDL-induced apoptosis in endothelial cells, and inhibition of apoptosis may be the molecular mechanism by which safflower exerts its endothelial protective effect. Bcl-2 and Bax are localized in the mitochondrial membrane and are involved in the permeability of the mitochondrial membrane to cytochrome C. The homodimer formed by Bax is a channel for cytochrome C to enter the cytoplasm from the mitochondria and promotes the release of cytochrome C. The heterodimer formed by Bcl-2 and Bax can antagonize the function of Bax and inhibit the release of cytochrome C. Cytochrome C, which enters the cytoplasm from the mitochondria, activates caspase-9 and eventually activates caspase-3 through a series of downstream cascade reactions, and the activated caspase-3 mediates apoptosis [53]. Under ox-LDL stimulation, Bcl-2 expression declined, Bax expression increased, and the Bcl-2/Bax ratio was significantly reduced in EAhy926 cells. In addition, TUNEL staining showed an increase in the number of TUNEL-positive cells in ox-LDL-stimulated EAhy926 cells, indicating that ox-LDL induced the apoptotic process in these cells. Meanwhile, the results of this study showed that safflower significantly inhibited the decrease in Bax expression, increase in Bcl-2 expression, and decrease in the Bax/Bcl-2 ratio in ox-LDL-stimulated EAhy926 cells, and the positive TUNEL staining in ox-LDL-stimulated EAhy926 cells was significantly reduced compared with the model group. It has been suggested that safflower has an inhibitory effect on ox-LDL-induced mitochondrial pathway apoptosis in endothelial cells, and antagonism of mitochondrial pathway apoptosis may be the molecular mechanism by which safflower exerts its protective effect on endothelial function.

The mitochondrial apoptotic pathway in endothelial cells is regulated by several signaling pathways, among which the SIRT1/FoxO1 pathway can sense energy changes and regulate the expression of mitochondrial pathway apoptotic genes such as Bcl-2 and Bax. SIRT1 is deacetylated and is inhibited under physiological conditions; however, when cells are stimulated by pathological factors such as ischemia and hypoxia and LPS, SIRT1 is activated, resulting in increased expression and deacetylation activity. Activated SIRT1 can deacetylate FoxO1, and the deacetylated FoxO1 enters the cytoplasm from the nucleus [54,55]; FoxO1 leaves the nucleus and loses its role in inhibiting Bax and promoting Bcl-2 expression, resulting in increased Bax expression and decreased Bcl-2 expression, which ultimately leads to apoptosis.

## 5. Conclusion

Using network pharmacology, the targets, mechanism of action, and associated signaling pathways of safflower in the therapy of CHD were assessed in this study. It was demonstrated that the effective compounds of safflower in the treatment of CHD may be dipalmitin, flavoxanthin, lignan, precarthamin, carthamone, quercetagetin, *β*-sitosterol, kaempferol, quercetin, baicalein, and 3'-methoxydaidzein. Possible targets include *VEGFA, STAT3, IL-6, IL-1B, MMP9, CCL2, CXCL8, TLR4, IL-10, ALB,* and putative pharmacological activities of other bioactive components linked to antioxidative stress, anti-apoptosis, and anti-inflammation. In addition, coronary artery disease can lead to the activation of the SIRT1/FoxO1 signaling pathway and activation of the apoptotic signaling pathway, and safflower can reverse this result. According to the findings, network pharmacology predictions could be useful in illustrating the molecular mechanisms of the Chinese herbal drug safflower in the treatment of CHD.

## Figures and Tables

**Table 1:** Comprehensive information on 34 safflower compounds. OB: oral bioavailability. DL: drug likeness.

Mol ID	Molecule Name	OB (%)	DL
MOL001281	L-Alpha-palmitin	26.66	0.22
MOL001771	Poriferast-5-en-3beta-ol	36.91	0.75
MOL001838	Dipalmitin	21.16	0.44
MOL002677	L-1,2-Dipalmitin	21.28	0.49
MOL002680	Flavoxanthin	60.41	0.56
MOL002681	Fluoranthene	24.7	0.18
MOL002684	Gamma-tocotrienol	20.3	0.53
MOL002687	Guanosine	21.43	0.21
MOL002694	4-[(E)-4-(3,5-dimethoxy-4-oxo-1-cyclohexa-2,5-dienylidene)but-2-enylidene]-2,6-dimethoxycyclohexa-2,5-dien-1-one	48.47	0.36
MOL002695	Lignan	43.32	0.65
MOL002698	Lupeol-palmitate	33.98	0.32
MOL002706	Phytoene	39.56	0.5
MOL002707	Phytofluene	43.18	0.5
MOL002708	Precarthamin	22	0.67
MOL002710	Pyrethrin II	48.36	0.35
MOL002712	6-Hydroxykaempferol	62.13	0.27
MOL002714	Baicalein	33.52	0.21
MOL002717	qt_carthamone	51.03	0.2
MOL002719	6-Hydroxynaringenin	33.23	0.24
MOL002721	Quercetagetin	45.01	0.31
MOL002735	Safflow-yellow-A	27.16	0.7
MOL002739	Tagetiin	28.34	0.78
MOL002757	7,8-Dimethyl-1H-pyrimido[5,6-g]quinoxaline-2,4-dione	45.75	0.19
MOL002759	Glyceryl pps	29.61	0.35
MOL002773	Beta-carotene	37.18	0.58
MOL002776	Baicalin	40.12	0.75
MOL000357	Sitogluside	20.63	0.62
MOL000359	Beta-sitosterol	36.91	0.75
MOL000422	Kaempferol	41.88	0.24
MOL000449	Stigmasterol	43.83	0.76
MOL000006	Luteolin	36.16	0.25
MOL000008	Apigenin	23.06	0.21
MOL000953	CLR	37.87	0.68
MOL000098	Quercetin	46.43	0.28

**Table 2:** The characteristics of top 10 hub genes.

Name	Betweenness	Closeness	Degree
*ALB*	0.05993046	0.78595318	172
*IL6*	0.03269393	0.7605178	162
*IL1B*	0.01853688	0.71428571	144
*VEGFA*	0.01291456	0.69117647	132
*STAT3*	0.01234645	0.66761364	122
*MMP9*	0.01367493	0.6545961	119
*TLR4*	0.01070128	0.66197183	118
*CCL2*	0.00746139	0.6545961	118
*CXCL8*	0.00663236	0.6456044	112
*IL10*	0.00652277	0.6420765	111

## Data Availability

The authors have presented all our main data in the article.
